# Asthma and Dietary Intake of Fish, Seaweeds, and Fatty Acids in Korean Adults

**DOI:** 10.3390/nu11092187

**Published:** 2019-09-11

**Authors:** Eun-kyung Kim, Se-Young Ju

**Affiliations:** Major in Food Science, College of Biomedical and Health Science, Konkuk University, Chungju, Chungbuk 27478, Korea

**Keywords:** asthma, fish, seaweeds, dietary fat, KNHANES

## Abstract

The dietary intake of fish and fatty acid may influence the risk of asthma, yet epidemiological research remains controversial and inconclusive. We examined the association between asthma and the dietary intake of fish, seaweeds, and fat in a Korean population, aged 19 to 64 years, using the data from the Korea National Health and Nutrition Examination Survey (KNHANES) 2013–2016 (*n* = 13,038). The prevalence of doctor-diagnosed asthma and medication prescribed asthma were 2.5% and 1.0%, respectively. The subjects with medication prescribed asthma had significantly lower consumption of seaweeds (*p* = 0.0110) and lower n3/n6 polyunsaturated fatty acid (PUFA) (*p* = 0.0275) as compared to subjects without medication prescribed asthma. Multiple logistic regression analysis showed that the odds ratio (OR) (95% confidence interval, CI) of doctor-diagnosed asthma in the highest quartile were 0.63 (0.41–0.97) and 0.66 (0.44–1.00) for fish and seaweeds respectively, compared to the lowest quartile after adjusting confounding factors. Furthermore, there were significant inverse associations between medication prescribed asthma and seaweeds [OR (95% CI) = 0.37 (0.19–0.70)], n-3 PUFA [OR (95% CI) = 0.43 (0.21–0.89)] and n3/n6 [OR (95% CI) = 0.47 (0.22–0.99)] intake after adjusting for confounding factors. These results suggest, that the higher consumption of fish and seaweed and the high ratio of n-3 to n-6 PUFA may be associated with a lower prevalence of asthma.

## 1. Introduction

The prevalence of asthma, one of the most common chronic diseases in children and young adults, has been increasing in the past few decades [1,2]. The worldwide prevalence of doctor diagnosed asthma and clinical asthma (treatment for asthma) in adults were 4.3% (ranging from 0.2% in China to 21.0% in Australia) and 4.5% (ranging from 1.0 in Vietnam to 21.5% in Australia), respectively [3]. In Korea, the prevalence of doctor diagnosed asthma was estimated to be within 0.7% to 2.0% between 1998 to 2008 [4].

Nutritional epidemiological studies have reported that the dietary intake of fruit [5,6], fish [7], n-3 polyunsaturated fatty acids (PUFA) [8,9], n-3/n-6 PUFA [7], oleic acids [10], and antioxidant nutrients [6,11] may be associated with asthma. Whether the dietary intake of fatty acid and fish can protect against asthma, however, remains controversial. A cross-sectional study found significant inverse associations between fish consumption and the high ratio of n-3/n-6 PUFA with asthma, among Japanese women [7]. A prospective cohort study (Coronary Artery Risk Development in Young Adults, CARDIA) of young adults in the United States showed that the incidence of asthma in participants in the highest quintile of long-chain n-3 PUFA intake significantly decreased by 54% compared to those in the lowest quintile [9]. However, the European Prospective Investigation into Cancer and nutrition (EPIC) Heidelberg cohort reported no protective effect of dietary fatty acids intake on asthma [10]. Additionally, many epidemiological and observational studies showed that maternal intake of fish and fatty acids during pregnancy was associated with asthma in infants or children resulting from those pregnancies [8,12,13,14].

In the Korean population, few studies have been performed on the associations between asthma and dietary factors, such as Kimchi consumption [15] and household food insecurity [16]. However, whether fish and fatty acid consumption is related to the prevalence of asthma has never been studied in Korea.

Therefore, the aim of this study was to identify the association between asthma and diet, including the intake of fish, seaweeds, and fatty acids in Korean adults, using the Korea National Health and Nutrition Examination Survey (KNHANES) data.

## 2. Methods

### 2.1. Study Population

**Figure 1:**
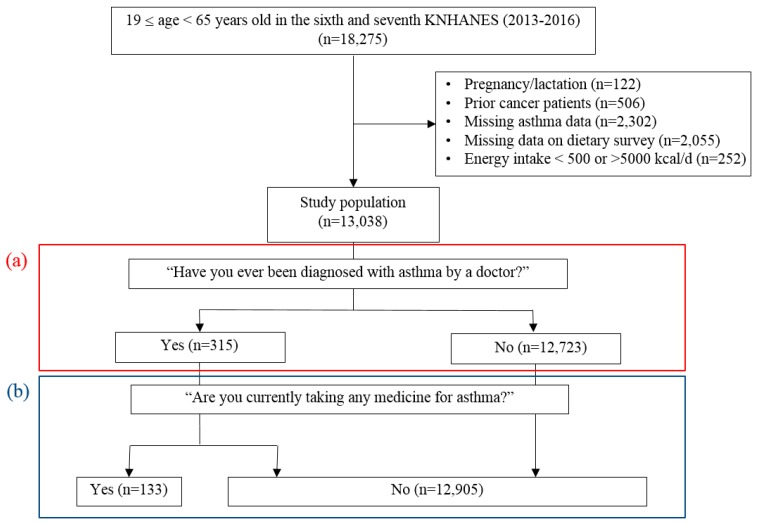
Flow chart of study population selection and the inclusion and exclusion criteria. (**a**) doctor-diagnosed asthma; (**b**) medication prescribed asthma.

This cross-sectional study used the sixth and seventh KNHANES (2013–2016) data conducted by the Korean Ministry of Health and Welfare. The KNHANES is a nationwide representative data based on stratified sampling design. Detailed information about the survey is available at http://knhanes.cdc.go.kr. The study population included men and women (aged 19 to 64 years old) who had completed a health examination and nutrition survey. We excluded a total of 5237 men and women; pregnant women (*n* = 122); prior cancer patients (e.g., liver, stomach, colorectal, uterine or cervical cancer) (*n* = 506); those with missing asthma data (*n* = 2302); those without 24 h dietary recall data (*n* = 2055); those with energy intake of less than 500 kcal/day or more than 5,000 kcal/day (*n* = 252). Thus, a total of 13,038 participants were used in the final analysis (Figure 1).

### 2.2. Definition of Asthma

Asthma was defined as doctor-diagnosed asthma and as medication prescribed asthma. In the health interview survey, the questionnaire contained questions on physician-diagnosed asthma and the medication used for asthma. We used the following questions in this study: “have you ever been diagnosed with asthma by a doctor?” and; “are you currently taking any medicine for asthma?”. Doctor-diagnosed asthma was described as the asthma diagnosed by a doctor. The participants were divided into a group without asthma (*n* = 23,723) and a group with asthma (*n* = 315), according to self-reported doctor-diagnosed asthma. Additionally, the participants were further divided into the group without asthma (*n* = 12,905) and the group with asthma (*n* = 133), based on the medication prescribed asthma medications.

### 2.3. Dietary Assessment

The dietary intakes of the participants were estimated by the 24-h dietary recall method. In this study, the food items were categorized into eight food groups including cereals/potatoes/sugar products, beans/nuts/seeds, meats and eggs, fishes and shellfishes, milk and dairy products, fruits and vegetable, mushrooms, and seaweeds. Dietary intakes of energy and 10 nutrients such as carbohydrate, protein, fat, calcium, iron, thiamin, riboflavin, niacin, vitamin C, vitamin A were estimated. Dietary fatty acids intakes including saturated fatty acid (SFA), monounsaturated fatty acids (MUFA), polyunsaturated fatty acid (PUFA), omega-3 (n3) fatty acids, omega-6 (n6) fatty acids, eicosapentaenoic acid (EPA), docosahexaenoic acid (DHA), cholesterol were calculated using 24-h dietary recall data in KNHANES. 

### 2.4. Covariates

Characteristics based on socioeconomic status, including age, sex (male and female), BMI (body mass index), residential area (urban and rural), education level (less than high school graduation, high school graduation, college graduate or higher), family income (low, moderately low, moderately high, high), were collected. Lifestyle characteristics including alcohol consumption (never, ≤1 drink/month, 2-4 drink/month, 2-3 drink/week, ≥4 drink/week), dietary supplement use (yes or no) and smoking status (current smokers, ex-smokers, nonsmokers) were collected.

### 2.5. Statistical Analysis

The PROC SURVEYFREQ procedure was used to examine differences in categorical variables according to the presence of asthma. The PROC SURVEYMEANS procedure was used to calculate the weighted mean and standard error of continuous variables. Statistical differences in the different asthma groups were analyzed with the PROC SURVEYREG procedure. Multivariate logistic regression was performed to estimate the odds ratios (ORs) and 95% confidence intervals (CIs) for asthma across the quartile of fish and shellfish, seaweeds, total fat and fatty acid intake—considering the lowest quartile as the reference. The adjusted variables were age, sex, residential area, household income, education level, smoking status, alcohol consumption, dietary supplement use, body mass index, energy intake, and fruit and vegetable intake. Statistical significance was considered as the data showed *p* < 0.05. All statistical analyses were performed using the SAS version 9.4 (SAS Institute, Cary, NC, USA).

## 3. Results

### 3.1. Characteristics Based on the Presence of Asthma

Characteristics of the study subjects according to the presence of asthma are shown in Table 1. Of the 13,038 eligible subjects, the prevalence of doctor-diagnosed asthma and medication prescribed asthma were 2.5% and 1.0%, respectively. The subjects with doctor-diagnosed asthma were likely to be younger than those without doctor-diagnosed asthma (*p* = 0.0335). However, the subjects with medication prescribed asthma were likely to be older, have higher BMI and have lower education level than those without medication prescribed asthma (*p* < 0.05). There were no significant differences in residential area, family income, alcohol consumption, dietary supplement use, smoking status in either the doctor-diagnosed asthma or the medication prescribed asthma groups. 

### 3.2. Daily Food and Nutrient Intake Based on the Presence of Asthma

Daily intakes of food and nutrients did not differ significantly in the doctor-diagnosed asthma group after adjustments for age, sex, residential area, household income, education level, smoking status, alcohol consumption, dietary supplement use, BMI, and energy intake were made. However, the subjects with doctor-diagnosed asthma (24.7 g/d) tended to consume fewer seaweeds compared to those without doctor-diagnosed asthma (17.4 g/d) (*p* = 0.0638). Interestingly, the subjects with medication-prescribed asthma had a significantly lower consumption of cereals/potatoes/sugar products (*p* = 0.0006) and seaweeds (*p* = 0.0110) than those without medication prescribed asthma. Additionally, in the subjects with medication-prescribed asthma, the mean daily intake of carbohydrate (*p* = 0.0195), iron (*p* = 0.0246), and n3/n6 (*p* = 0.0275) were lower compared to the subjects without medication prescribed asthma (Table 2). 

### 3.3. Association between Dietary Intake and Asthma 

Adults in the highest quartiles showed significantly decreased prevalence of doctor-diagnosed asthma based on the dietary intake of certain foods. Specifically, we found a 37% reduction for fish [OR (95% CI) = 0.63 (0.41–0.97), *p* for trend = 0.0637], intake and a 34% reduction for seaweeds [OR (95% CI) = 0.66 (0.44–1.00), *p* for trend = 0.0300] intake, after adjustment for confounding factors. In the subjects with medication-prescribed asthma, there was a significant negative association between seaweeds and medication prescribed asthma [OR (95% CI) = 0.37 (0.19–0.70), *p* for trend < 0.0001] (Table 3).

Multiple logistic regression analysis showed a significant negative association between specific dietary fatty acid and asthma (Table 4). Medication prescribed asthma was inversely associated with n-3 PUFA [OR (95% CI) = 0.43 (0.21–0.89), *p* for trend = 0.4776] and n3/n6 [OR (95% CI) = 0.47 (0.22–0.99), *p* for trend = 0.0563] intake—after adjusting for confounding factors.

## 4. Discussion

In this cross–sectional study from KNHANES (2013–2016), we examined the relationship between asthma and dietary intake of fish, seaweeds, and fatty acids among Korean adults. The subjects with medication prescribed asthma were found to be older, have higher BMI and have lower education level than those without medication–prescribed asthma. For the daily intake of foods and nutrients, the subjects with doctor–diagnosed asthma tended to consume fewer seaweeds than those without doctor-diagnosed asthma. The subjects with medication prescribed asthma had a significantly lower consumption of cereals/potatoes/sugar products and seaweeds than those without medication prescribed asthma. We found a high consumption of fish, seaweeds, n-3 PUFA, and a high ratio of n3/n6 PUFA were inversely associated with the prevalence of asthma.

Asthma is a chronic inflammatory disease that is closely associated with environmental and dietary factors. Recently, there has been growing evidence suggesting that the dietary intake of vitamin C, E, D, and n-3 PUFA play an important role in modulating morbidity caused by asthma and allergic diseases [17]. Biochemical studies show, that anti-inflammatory precursors such as resolvins, protectins, and maresins are converted from omega-3(n-3) fatty acid, while arachidonic acid (n-6 PUFA)-derived prostaglandins and leukotrienes act as pro-inflammatory mediators [8]. Since high intake of fish and seafood may help reduce the prevalence of asthma, many epidemiological and clinical studies have evaluated the relationship between asthma and the intake of fish oil—especially n-3 PUFA. Miyamoto et al. [7] reported that fish consumption and the ratio of n-3/n-6 PUFA, had significant inverse relationships with the prevalence of asthma in Japanese young adults. Similar to our findings, this study reported as well that the ratio of n-3/n-6 PUFA in the highest tertile showed a significant decrease in prevalence of asthma. Another study of the 20-year follow-up longitudinal analysis among American young adults showed as well that intake of long-chain n-3 PUFAs inversely associated with the prevalence of asthma. Several studies examined the relationship between maternal fish intake during pregnancy and the resulting atopy and asthma in children during infancy. These studies reported that fish intake during pregnancy was associated with a protective effect on the incidence of allergic diseases including atopy, eczema, and asthma in early infancy [18,19,20,21]. A recent systematic review on maternal dietary intake and asthma showed that, offspring of mothers with diets rich in fruits and vegetables, fish, or Mediterranean diets (rich in fruits and vegetables, and low in refined grains and saturated fat) were associated with a lower risk of asthma [22,23]. Additionally, several studies reported the beneficial effects of fish intake during childhood, showing decreased rate or prevention of asthma and atopic diseases. The studies of Hodge et al. [24] and Takemura et al. [25] reported the inverse association between fish consumption and the prevalence of asthma in children aged between six to 15 years. The recent meta-analysis studying the association between fish intake and risk of childhood asthma found that fish intake in infants was negatively related to the incidence of asthma [26]. However, other studies have shown inconsistent or negative results of fish and n-3 PUFA intakes on the risk of asthma. Another prospective study from Nagel and Linseisen, did not find any association between the dietary intake of n-3 PUFA, α-linolenic acid, eicosapentaenoic acid (EPA), and docosahexaenoic acid (DHA) and asthma [10]. Similarly, a study where 60,774 mother-child pairs participated in 18 European and US birth cohort studies negated the beneficial effects of intake of fish and seafood during pregnancy on the risk of allergic diseases including asthma, wheezing, and allergic rhinitis in children up to eight years old [27]. A case-control study reported that intake of n-3 PUFA did not show any significant differences in the effects between asthmatic and non-asthmatic adults [28]. Moreover, several birth cohort studies failed to report a protective effect of maternal dietary intake of n-3 PUFA or n-3 in cord blood on asthma risk in childhood [29,30,31].

On the contrary, reports on dietary intake of monounsaturated fatty acids and trans-fatty acids show a positive correlation to the risk of asthma and other allergic diseases [32,33,34]. Dunder et al. [35] reported as well that children with atopy were positively associated with higher intake of margarine and lower intake of butter than those with non-atopy. Similarly, Nagel and Linseisen [10] reported as well that high dietary intakes of oleic acid (C18:1) and margarine were positively associated with the risk of asthma in adults. Omega-6 (n-6) PUFAs in margarine and vegetable oil are converted into arachidonic acid, which can produce proinflammatory metabolites such as prostaglandins and leukotrienes [8]. Therefore, it is still controversial whether the intakes of n-3 PUFAs and fish can reduce the risk of asthma or whether the intake of n-6 PUFAs can increase the risk of asthma.

This study has some limitations. First, the prevalence of asthma could not be confirmed due to the self-administered survey as opposed to standardized measures. Second, we could not assess an accurate causal association between the intake of fish and n-3 PUFAs and the risk of asthma because of the cross-sectional design. Third, we could not calculate the actual intake of fish and n-3 PUFAs from the participants’ diet, because we could only collect the one-day 24-h recall data. However, this result can be generalized to the Korean people since a nationwide representative population-based sample of Korean origin was used. Moreover, this result might have an important implication for public health and further studies. Based on this study, further clinical research is required to determine the relationship between asthma and diet with certainty.

## 5. Conclusions

There are significantly inverse associations between consumption of fish, seaweeds, and ratio of n3/n6 PUFA and asthma in Korean adults. These results suggest that high consumption of fish, seaweeds, and foods rich in n3 PUFA may provide benefits to asthma. Further studies are needed to confirm the mechanisms related to the beneficial effects of fish and seaweeds and PUFA against asthma. 

## Figures and Tables

**Table 1:** General characteristics of the subjects according to asthma.

Variables	Doctor-Diagnosed Asthma			Medication-Prescribed Asthma		
	Without (*n* = 12,723)	With (*n* = 315)	*p*-Value	Without (*n* = 12,905)	With (*n* = 133)	*p*-Value
Sex						
Male	5113 (50.6)	105 (45.1)	0.1049	5175 (50.5)	43 (45.0)	0.2904
Female	7610 (49.4)	210 (54.9)		7730 (49.5)	90 (55.0)	
Age (years)	41.2 ± 0.2	39.2 ± 1.0	0.0335	41.1 ± 0.2	44.9 ± 1.4	0.0063
BMI (kg/m^2^)	23.8 ± 0.0	24.1 ± 0.2	0.1387	23.8 ± 0.0	24.8 ± 0.4	0.0106
Residential area						
Urban	10611 (85.7)	256 (84.2)	0.4917	10765 (85.7)	102 (81.4)	0.2037
Rural	2112 (14.3)	59 (15.8)		2140 (14.3)	31 (18.6)	
Education level						
<High school graduate	6170 (43.5)	161 (42.3)	0.8637	6241 (43.3)	90 (58.0)	0.0185
High school graduate	2090 (17.7)	51 (19.0)		2129 (17.8)	12 (11.8)	
≥College graduate	4429 (38.8)	102 (38.7)		4500 (38.9)	31 (30.1)	
Household income						
Low	1192 (9.0)	42 (11.2)	0.1535	1210 (9.0)	24 (15.7)	0.0993
Moderately low	3142 (24.3)	65 (21.3)		3176 (24.2)	31 (22.6)	
Moderately high	3963 (31.5)	111 (36.4)		4033 (31.6)	41 (32.2)	
High	4389 (35.3)	97 (31.0)		4449 (35.2)	37 (29.5)	
Alcohol consumption						
Never	1786 (13.2)	59 (16.4)	0.4215	1814 (13.2)	31 (21.5)	0.1548
≤1 drink/month	3902 (32.1)	97 (33.2)		3960 (32.2)	39 (31.0)	
2–4 drink/month	3192 (29.3)	69 (28.7)		3241 (29.3)	20 (21.1)	
2–3 drink/week	2032 (18.7)	47 (17.5)		2060 (18.6)	19 (19.3)	
≥4 drink/week	747 (6.7)	12 (4.2)		752 (6.6)	7 (7.1)	
Dietary supplement use						
Yes	5861 (44.4)	133 (41.1)	0.2846	5938 (44.3)	56 (40.8)	0.4951
Smoking status						
Current smokers	2172 (25.8)	54 (25.0)	0.6101	2202 (25.8)	24 (29.4)	0.8019
Ex-smokers	379 (4.4)	5 (2.9)		382 (4.4)	2 (3.5)	
Nonsmokers	7974 (69.8)	207 (72.0)			81 (67.1)	

Data are n(%) or mean ± SE.

**Table 2:** Daily food and nutrient intakes of the subjects according to asthma.

Variables	Doctor-Diagnosed Asthma			Medication-Prescribed Asthma		
	Without (*n* = 12,723)	With (*n* = 315)	*p*-Value	Without (*n* = 12,905)	With (*n* = 133)	*p*-Value
Food intake (g)						
Cereals/potatoes/sugar products	349.3 ± 2.3	333.2 ± 11.3	0.2961	349.1 ± 2.2	321.7 ± 16.1	0.0006
Beans/nuts/seeds	44.0 ± 0.9	38.3 ± 4.1	0.5653	43.9 ± 0.9	41.5 ± 7.2	0.6520
Meats and eggs	146.8 ± 1.9	132.7 ± 10.5	0.9874	146.6 ± 1.9	127.6 ± 17.5	0.8904
Fishes and shellfishes	98.2 ± 1.9	83.1 ± 10.2	0.5890	97.9 ± 1.9	90.0 ± 20.8	0.8761
Fishes	27.6 ± 0.7	22.4 ± 3.3	0.0923	27.5 ± 0.7	27.1 ± 5.8	0.5785
Milk and dairy products	88.9 ± 1.8	110.2 ± 14.8	0.2664	89.1 ± 1.8	118.4 ± 29.9	0.3723
Fruit and vegetable	518.1 ± 4.6	494.5 ± 24.9	0.8628	517.3 ± 4.6	534.1 ± 39.9	0.9614
Mushrooms	6.7 ± 0.3	4.5 ± 0.8	0.0964	6.7 ± 0.3	4.9 ± 1.4	0.3942
Seaweeds	24.7 ± 1.1	17.4 ± 3.3	0.0638	24.6 ± 1.1	13.5 ± 4.1	0.0110
Energy and nutrient intake						
Energy (kcal)	2128.8 ± 9.8	2028.3 ± 58.3	0.0705	2127.0 ± 9.7	2056.5 ± 97.6	0.6463
Carbohydrate (g)	312.0 ± 1.4	302.7 ± 8.3	0.7192	311.8 ± 1.4	302.2 ± 12.7	0.0195
Protein (g)	75.2 ± 0.4	71.6 ± 3.0	0.4817	75.2 ± 0.4	74.3 ± 5.4	0.3215
Fat (g)	50.6 ± 0.4	47.6 ± 2.0	0.7848	50.5 ± 0.4	47.2 ± 3.3	0.3143
Calcium (mg)	501.8 ± 3.3	496.7 ± 25.5	0.4282	501.5 ± 3.3	524.7 ± 46.1	0.5543
Iron (mg)	17.7 ± 0.2	16.0 ± 0.6	0.1972	17.6 ± 0.2	16.1 ± 1.0	0.0246
Thiamin (mg)	2.1 ± 0.0	2.0 ± 0.1	0.9728	2.1 ± 0.0	2.1 ± 0.1	0.5263
Riboflavin (mg)	1.5 ± 0.0	1.5 ± 0.1	0.1498	1.5 ± 0.0	1.6 ± 0.2	0.1721
Niacin (mg)	17.4 ± 0.1	16.5 ± 0.7	0.8362	17.4 ± 0.1	17.2 ± 1.4	0.6666
Vitamin C (mg)	100.5 ± 1.5	95.6 ± 7.5	0.8166	100.3 ± 1.5	110.3 ± 13.6	0.8952
Vitamin A (μgRE)	751.9 ± 11.1	780.7 ± 75.2	0.6336	752.1 ± 11.1	798.2 ± 160.8	0.6804
Fatty acids						
SFA (g)	14.7 ± 0.1	14.3 ± 0.7	0.3194	14.7 ± 0.1	14.5 ± 1.3	0.1252
MUFA (g)	16.2 ± 0.1	15.3 ± 0.8	0.7624	16.2 ± 0.1	15.4 ± 1.2	0.1925
PUFA (g)	12.2 ± 0.1	11.4 ± 0.6	0.9407	12.2 ± 0.1	11.0 ± 0.8	0.9465
n-3 (g)	1.7 ± 0.0	1.5 ± 0.1	0.8966	1.7 ± 0.0	1.5 ± 0.1	0.6403
n-6 (g)	10.6 ± 0.1	9.8 ± 0.5	0.9783	10.6 ± 0.1	9.6 ± 0.7	0.8517
n3/n6	0.18 ± 0.00	0.17 ± 0.01	0.0708	0.18 ± 0.00	0.16 ± 0.01	0.0275
EPA (mg)	108.2 ± 2.6	96.2 ± 15.7	0.9384	107.6 ± 2.6	135.9 ± 37.0	0.3860
DHA (mg)	187.7 ± 5.2	152.1 ± 22.5	0.5155	186.6 ± 5.1	205.4 ± 50.5	0.5818
Cholesterol (mg)	283.5 ± 2.9	287.6 ± 19.0	0.4395	283.5 ± 2.8	291.7 ± 31.0	0.3442

Fish: daily fish intake except for salted and fermented seafood, adjusted for age, sex, residential area, education level, household income, alcohol consumption, dietary supplement use, smoking status, body mass index, and energy intake.

**Table 3:** Association between fish and seaweeds intake and asthma.

Variable	Range (g)	Median (g)	Doctor-Diagnosed Asthma			Medication-Prescribed Asthma		
			Prevalence (%)	Crude OR (95% CI)	Adjusted OR (95% CI)	Prevalence (%)	Crude OR (95% CI)	Adjusted OR (95% CI)
Fish and shellfish								
Q1	<4.07	0	94/3259 (2.88)	1	1	41/3259 (1.26)	1	1
Q2	4.07–35.20	14.82	78/3260 (2.39)	0.82 (0.60–1.13)	0.90 (0.62–1.32)	32/3260 (0.98)	0.71 (0.42–1.20)	0.77 (0.41–1.44)
Q3	35.21–125.14	67.05	71/3260 (2.18)	0.82 (0.58–1.17)	0.75 (0.49–1.15)	35/3260 (1.07)	0.93 (0.56–1.55)	0.71 (0.38–1.34)
Q4	>125.14	233.34	72/3259 (2.21)	0.77 (0.54–1.10)	0.85 (0.57–1.27)	25/3259 (0.77)	0.55 (0.31–0.98)	0.57 (0.29–1.12)
*P* for trend				0.2705	0.6894		0.0501	0.1005
Fish								
Q1	0	0	135/4782 (2.82)	1	1	51/4782 (1.07)	1	1
Q2	<2.49	1.09	43/1737 (2.48)	0.73 (0.49–1.11)	0.78 (0.49–1.24)	17/1737 (0.98)	0.78 (0.42–1.43)	0.80 (0.38–1.69)
Q3	2.49–24.01	7.46	74/3260 (2.27)	0.65 (0.47–0.91)	0.71 (0.48–1.04)	36/3260 (1.10)	0.71 (0.45–1.12)	0.60 (0.34–1.07)
Q4	>24.01	69.32	63/3259 (1.93)	0.71 (0.51–0.99)	0.63 (0.41–0.97)	29/3259 (0.89)	0.91 (0.54–1.53)	0.77 (0.40–1.50)
*P* for trend				0.1617	0.0637		0.9453	0.6532
Seaweeds								
Q1	0	0	141/5627 (2.51)	1	1	62/5627 (1.10)	1	1
Q2	<1.00	0.50	27/896 (3.01)	0.82 (0.50–1.34)	0.78 (0.43–1.40)	10/896 (1.12)	0.84 (0.38–1.84)	0.82 (0.30–2.24)
Q3	1.01–7.47	3.07	75/3256 (2.30)	0.87 (0.64–1.19)	0.95 (0.66–1.36)	34/3256 (1.04)	0.88 (0.56–1.41)	0.99 (0.57–1.75)
Q4	>7.47	28.84	72/3259 (2.21)	0.72 (0.53–0.98)	0.66 (0.44–1.00)	27/3259 (0.83)	0.52 (0.32–0.85)	0.37 (0.19–0.70)
*P* for trend				0.0346	0.0300		0.0018	<0.0001

Doctor-diagnosed asthma: self-reported doctor diagnosis of asthma. Medication prescribed asthma: prescribed oral and inhaled asthma medications in the past 12 months. Fish: daily fish intake except for salted and fermented seafood. Adjusted for age, sex, residential area, education level, household income, alcohol consumption, dietary supplement use, smoking status, body mass index, energy intake and fruit and vegetable intake.

**Table 4:** Association between specific types of dietary fat and asthma.

Variable	Range (g)	Median (g)	Doctor-Diagnosed Asthma			Medication-Prescribed Asthma		
			Prevalence (%)	Crude OR (95% CI)	Adjusted OR (95% CI)	Prevalence (%)	Crude OR (95% CI)	Adjusted OR (95% CI)
Total fat								
Q1	<24.52	16.94	89/3259 (2.73)	1	1	42/3259 (1.29)	1	1
Q2	24.52–39.69	32.00	73/3260 (2.24)	0.79 (0.55–1.14)	0.75 (0.48–1.17)	32/3260 (0.98)	0.78 (0.46–1.32)	0.76 (0.38–1.50)
Q3	39.70–60.98	48.90	83/3260 (2.55)	0.90 (0.64–1.26)	0.92 (0.59–1.43)	31/3260 (0.95)	0.74 (0.47–1.17)	0.96 (0.48–1.93)
Q4	>60.98	83.53	70/3259 (2.15)	0.80 (0.55–1.16)	1.04 (0.60–1.81)	28/3259 (0.86)	0.75 (0.44–1.27)	1.61 (0.65–3.99)
*P* for trend				0.3792	0.6269		0.3702	0.2090
SFA								
Q1	<6.36	4.21	783259 (2.39)	1	1	41/3259 (1.26)	1	1
Q2	6.36–11.02	8.67	82/3260 (2.52)	1.18 (0.82–1.69)	1.21 (0.78–1.89)	26/3260 (0.80)	0.67 (0.38-1.20)	0.59 (0.83-1.15)
Q3	11.03-17.73	13.96	77/3260 (2.36)	1.15 (0.80-1.65)	1.33 (0.84-2.11)	38/3260 (1.17)	1.05 (0.66-1.68)	1.59 (0.83-3.08)
Q4	>17.73	25.05	78/3259 (2.39)	1.11 (0.77-1.60)	1.63 (0.97-2.73)	28/3259 (0.86)	0.80 (0.46-1.37)	1.58 (0.74-3.38)
*P* for trend				0.7875	0.0743		0.7171	0.0871
MUFA								
Q1	<6.90	4.55	87/3259 (2.67)	1	1	42/3259 (1.29)	1	1
Q2	6.90-12.04	9.48	79/3260 (2.42)	0.98 (0.68-1.39)	0.99 (0.63-1.55)	31/3260 (0.95)	0.89 (0.52-1.50)	1.14 (0.57-2.27)
Q3	12.05–19.76	15.24	78/3260 (2.39)	0.91 (0.63–1.32)	0.87 (0.54–1.40)	31/3260 (0.95)	0.75 (0.46–1.23)	0.92 (0.41–2.08)
Q4	>19.76	27.87	71/3259 (2.18)	0.87 (0.60–1.26)	1.05 (0.57–1.93)	29/3259 (0.89)	0.84 (0.50–1.43)	1.77 (0.66–4.76)
*P* for trend				0.4361	0.8685		0.5791	0.2505
PUFA								
Q1	<5.57	3.76	95/3259 (2.92)	1	1	48/3259 (1.47)	1	1
Q2	5.57–9.24	7.36	64/3260 (1.96)	0.67 (0.46–0.97)	0.72 (0.45–1.16)	27/3260 (0.83)	0.56 (0.33–0.94)	0.58 (0.28–1.18)
Q3	9.25–14.92	11.71	87/3260 (2.67)	1.04 (0.74–1.46)	1.09 (0.70–1.69)	30/3260 (0.92)	0.85 (0.52–1.40)	0.86 (0.42–1.75)
Q4	>14.92	21.0	69/3259 (2.12)	0.74 (0.51–1.07)	1.10 (0.66–1.84)	28/3259 (0.86)	0.55 (0.33–0.93)	0.85 (0.37–1.99)
*P* for trend				0.3328	0.3753		0.0803	0.9308
n–3 PUFA								
Q1	<0.65	0.41	91/3259 (2.79)	1	1	49/3259 (1.50)	1	1
Q2	0.65–1.19	0.90	77/3260 (2.36)	0.82 (0.58–1.16)	0.86 (0.57–1.29)	28/3260 (0.86)	0.55 (0.32–0.93)	0.62 (0.31–1.22)
Q3	1.20–2.10	1.56	81/3260 (2.48)	0.80 (0.56–1.14)	0.82 (0.53–1.26)	27/3260 (0.83)	0.47 (0.27–0.82)	0.43 (0.21–0.89)
Q4	>2.10	3.07	66/3259 (2.03)	0.74 (0.51–1.10)	0.90 (0.57–1.40)	29/3259 (0.89)	0.63 (0.37–1.07)	0.71 (0.33–1.51)
*P* for trend				0.1918	0.7609		0.2290	0.4776
n–6 PUFA								
Q1	<4.65	3.09	93/3259 (2.85)	1	1	48/3259 (1.47)	1	1
Q2	4.65–7.85	6.24	72/3260 (2.21)	0.82 (0.56–1.19)	0.92 (0.58–1.45)	28/3260 (0.86)	0.64 (0.38–1.09)	0.69 (0.35–1.36)
Q3	7.86–12.93	10.04	81/3260 (2.48)	0.99 (0.70–1.41)	1.03 (0.65–1.63)	29/3260 (0.89)	0.79 (0.47–1.31)	0.82 (0.38–1.74)
Q4	>12.93	18.30	69/3259 (2.12)	0.75 (0.52–1.09)	1.07 (0.63–1.82)	28/3259 (0.86)	0.54 (0.32–0.89)	0.80 (0.32–1.98)
*P* for trend				0.2015	0.6872		0.0305	0.7295
n–3/n–6								
Q1	<0.10	0.08	92/3259 (2.82)	1	1	45/3259 (1.38)	1	1
Q2	0.10–0.13	0.12	68/3259 (2.09)	0.78 (0.54–1.15)	0.92 (0.59–1.43)	29/3259 (0.89)	0.60 (0.35–1.02)	0.61 (0.32–1.16)
Q3	0.14–0.19	0.16	75/3260 (2.30)	0.89 (0.63–1.27)	1.01 (0.66–1.58)	33/3260 (1.01)	0.75 (0.45–1.25)	0.69 (0.36–1.31)
Q4	>0.19	0.30	80/3259 (2.45)	0.92 (0.65–1.30)	0.91 (0.58–1.43)	26/3259 (0.80)	0.59 (0.33–1.06)	0.47 (0.22–0.99)
*P* for trend				0.9761	0.7471		0.1475	0.0563
Cholesterol								
Q1	<93.07	48.68	97/3259 (2.98)	1	1	45/3259 (1.38)	1	1
Q2	93.07–202.34	142.84	62/3260 (1.90)	0.61 (0.42–0.90)	0.64 (0.41–0.99)	33/3260 (1.01)	0.76 (0.48–1.19)	0.84 (0.48–1.49)
Q3	202.35–361.02	272.54	81/3260 (2.48)	0.86 (0.61–1.21)	1.05 (0.73–1.51)	27/3260 (0.83)	0.60 (0.35–1.04)	0.72 (0.37–1.42)
Q4	>361.02	534.53	75/3259 (2.30)	0.87 (0.61–1.24)	0.85 (0.56–1.31)	28/3259 (0.86)	0.88 (0.53–1.46)	0.96 (0.50–1.85)
*P* for trend				0.9132	0.9354		0.8583	0.9555

Doctor–diagnosed asthma: self–reported doctor diagnosis of asthma. Medication prescribed asthma: prescribed oral and inhaled asthma medications in the past 12 months. Adjusted for age, sex, residential area, education level, household income, alcohol consumption, dietary supplement use, smoking status, body mass index, energy intake and fruit and vegetable intake.
